# The Impact of the COVID-19 Pandemic on the Clinical and Epidemiological Profile of Severe Acute Respiratory Infection in Bahia, Brazil: A Comparative Analysis of Pre- and Post-Pandemic Trends

**DOI:** 10.3390/v17030389

**Published:** 2025-03-09

**Authors:** Káriton Magalhães Bronze, Uener Ribeiro dos Santos, Galileu Barbosa Costa, Anaiá da Paixão Sevá, Maíra Guimarães Kersul, Cathianne Sacramento Pinto, George Rego Albuquerque, Ana Paula Melo Mariano, Sandra Rocha Gadelha

**Affiliations:** 1 Laboratório de Farmacogenômica e Epidemiologia Molecular, Universidade Estadual de Santa Cruz, Ilhéus 45662-900, Bahia, Brazil; karitonbronze@hotmail.com (K.M.B.); gralbu@uesc.br (G.R.A.); paulamariano@uesc.br (A.P.M.M.); srgmello@uesc.br (S.R.G.); 2 Programa de Pós-Graduação em Ciências da Saúde (PPGCS), Universidade Estadual de Santa Cruz, Ilhéus 45662-900, Bahia, Brazil; 3 Faculdade Ages de Medicina de Irecê, Colegiado de Ciências Biológicas e da Saúde, Rua Atacadão, Irecê 44900-000, Bahia, Brazil; 4 Programa de Pós-Graduação em Ciência Animal (PPGCA), Universidade Estadual de Santa Cruz, Ilhéus 45662-900, Bahia, Brazil; apseva@uesc.br (A.d.P.S.); mgkersul@uesc.br (M.G.K.); 5 Secretaria de Saúde do Estado da Bahia, Salvador 45652-050, Bahia, Brazil; caca.enfermeira@hotmail.com

**Keywords:** SARS-CoV-2, influenza virus, respiratory syncytial virus, seasonality, symptoms, SARI

## Abstract

In recent years, the incidence of Severe Acute Respiratory Infection (SARI) has increased due to the emergence of SARS-CoV-2. However, the impact of the COVID-19 pandemic extends beyond mortality rates. Recent analyses suggest that the introduction and spread of SARS-CoV-2 have significantly affected the epidemiology of other key respiratory viruses, such as influenza virus (FLUV), respiratory syncytial virus (RSV), and rhinovirus (RV). These changes raise new questions about the dynamics and incidence of post-COVID-19 respiratory infections, as well as potential alterations in symptom profiles and clinical outcomes. In this study, we analyzed data from the Epidemiological Surveillance Information System of Respiratory Viral Agents (SIVEP-Gripe), established by the Brazilian Ministry of Health, to examine the profile of SARI before and during the COVID-19 pandemic in Brazil. Our data reveal a distinct epidemiological pattern, with a significant decrease in FLUV notifications during the pandemic, accompanied by peaks in RSV and RV cases in late 2020. Additionally, there was a shift in the age distribution of RSV and other viral infections, with individuals infected during the pandemic being older than those infected before the pandemic. Interestingly, the introduction and spread of SARS-CoV-2 in Bahia State resulted in a reduction in the frequency of symptoms associated with non-SARS-CoV-2 SARI, without altering clinical outcomes. Our findings suggest that the circulation of SARS-CoV-2 has contributed to a clinical and epidemiological shift, particularly for FLUV, RSV, and other viruses, marked by a reduction in symptoms such as fever, dyspnea, respiratory distress, and the need for ventilatory support. The underlying mechanisms driving these changes remain unclear. These insights are crucial for public health authorities and policymakers to refine surveillance strategies and enhance control measures for respiratory viruses, particularly those causing SARI.

## 1. Introduction

Influenza-like illness (ILI) refers to a clinical syndrome characterized by the presence of fever (≥38 °C) and either cough or sore throat, in the absence of a known etiology other than influenza [1]. A clinical diagnosis of influenza is initially considered a diagnosis of ILI until further confirmation by laboratory testing [1]. Since 2011, the World Health Organization (WHO) has established the definition of severe acute respiratory infection (SARI) for global surveillance purposes [2]. This updated definition applies to all age groups to streamline implementation, omitting terms like “shortness of breath” and “breathing difficulty,” while adding “history of fever” and extending the symptom onset period to 10 days. SARI has been now defined as an acute respiratory illness characterized by a history of fever and cough, with symptoms beginning within the last 10 days and requiring hospitalization [2,3]. This case definition helps track severe influenza-related diseases and assess their burden.

Influenza viruses (FLUVs) are a significant public health concern and etiological agents of SARI, resulting in substantial socioeconomic burdens, with less than 10% of the world population routinely vaccinated [4]. Influenza virus types A (FLUAV) and B (FLUBV) exhibit seasonal epidemiology, with an attack rate of 5–10% in adults and 20–30% in children [5]. The WHO estimates approximately 5 million cases of severe illness caused by FLUV, with up to 650,000 respiratory deaths annually [6].

In Brazil, more than 90% of severe disease caused by FLUVs among adults required hospitalization, with case-fatality rates reaching as high as 15% [7]. Additionally, case-fatality rates rise to 24.6% in older adults, with higher rates observed in male and individuals of brown ethnicity [8]. Peaks of FLUV activity typically occur during May to August, or during March to June, with high mortality rates observed in the Southern region of Brazil [9,10]. Although a vaccine against circulating FLUV is available in Brazil, updated annually, and administered seasonally before fall and winter, many FLUV cases are still eventually detected as a result of antigenic drift that may result in poor matching [10], highlighting the challenges of preventing ILI and SARI episodes. In 2009, after the WHO declared a pandemic H1N1, the Brazilian Ministry of Health created the Respiratory Syndrome Surveillance System (known as SIVEP-Gripe) to monitor FLUV, with consequently other respiratory viruses being included into the surveillance network system [11,12].

Other respiratory viruses such as respiratory syncytial virus (RSV), rhinovirus (RV) types A and B, human metapneumovirus (HMPV), adenovirus (AdV), human bocavirus (HBoV), and parainfluenza virus (PIV) can also cause SARI in both adults and children [10,13]. However, the overall frequency has been reported to be very low when compared to severe acute respiratory syndrome coronavirus 2 (SARS-CoV-2) and FLUV [14]. In December 2019, after SARS-CoV-2 emerged in China and rapidly spread worldwide, causing an unprecedented public health threat, a significantly increasing number of severe cases have been reported, therefore impacting the epidemiology of other respiratory viruses [15,16,17].

In Brazil, 39 million cases of COVID-19 have been reported, with 715,000 confirmed deaths. The northeast region was the third most affected, with 7.6 million cases and 137,000 deaths [18]. Bahia, the fourth most populous state of Brazil, located in the northeast region (11°24′35.5464″ S, 41°16′51.0852″ W), with a low Human Development Index (HDI) and a per capita income of approximately USD 200 was the most affected state in the northeast region, reporting a total of 1.8 million cases and more than 32,000 confirmed deaths [19].

The non-pharmacological interventions used to counteract the spread of SARS-CoV-2 have reduced the transmission of other respiratory viruses with similar routes of transmission [17,20], also reducing their genetic diversity due to decreasing in viral circulation [15]. Indeed, reduced circulation of RSV, FLUV, HMPV, and AdV has been reported in the USA during the COVID-19 pandemic [16]. The presence of SARS-CoV-2 appears to also change the clinical course of severe disease caused by other respiratory viruses. A study has reported a 3.8 and 16-fold decrease in ICU admission among children caused by RSV and FLUV, respectively [17]. Complications due to bronchitis have also decreased by 3- and 3.2-fold before (2018–2019) and during (2020–2021) the COVID-19 pandemic, respectively [17]. In Korea, the COVID-19 has changed the epidemiology of non-respiratory diseases, such as gastrointestinal infections, Kawasaki disease, and Hepatitis [21].

In Brazil, including Bahia, FLUV and other respiratory viruses pose a significant public health challenge, substantially impacting morbidity and mortality rates. Between 2021 and 2023, the predominant FLUV strain circulating in the country included A/H1N1pdm09, A/H3N2, and B/Victoria [22]. Despite community-related variables, social factors such as sanitation, inequalities, and economic conditions play a crucial role in shaping the epidemiology of respiratory viruses [23]. Previous studies have shown that community variables influence the dynamics of SARS-CoV-2 infection across cities of Bahia [24] and that occupational status can affect the risk of SARS-CoV-2 infections [25]. However, no study has yet been conducted to assess the impact of the COVID-19 pandemic on the epidemiology of other respiratory viruses in Bahia.

Here, we used data from the Epidemiological Surveillance Information System of respiratory viral agents (SIVEP-Gripe), reported by the Brazilian Ministry of Health, to analyze the profile of SARI and severe respiratory disease before and during the COVID-19 pandemic in Bahia State, Brazil.

## 2. Materials and Methods

### 2.1. Ethical Considerations

This study was submitted to the Research Ethics Committee of the Universidade Estadual de Santa Cruz (CEP/UESC) and approved under protocol number CAAE:52140621.2.0000.5526. Patient confidentiality was maintained, and no patient identification information was assessed.

### 2.2. Study Design and Data Curation

We conducted an observational, ecological analytical study using secondary data from the SIVEP-Gripe database (https://sivepgripe.saude.gov.br/sivepgripe/login.html?0, accessed on 9 January 2022). Official data from Bahia State were collected for two periods: January–December 2019 (pre-COVID-19 pandemic) and January–December 2020 (the first year of the COVID-19 pandemic). The inclusion criteria comprised individuals with fixed residency in Bahia State and registered in the SIVEP-Gripe system between January 2019 and December 2020, who tested positive for respiratory viral infections by PCR assay and had available clinical and epidemiological data. The exclusion criteria comprised individuals who tested negative for respiratory viral infections by PCR or had an inconclusive result, individuals from other States, and those with incomplete data.

The collected variables included the number of SIVEP-Gripe notifications, samples tested for respiratory viruses, demographic information (gender, age, and self-reported ethnicity), clinical symptoms, comorbidities, outcomes, and the need for ventilatory support. Clinical conditions and comorbidities included in the study were those listed in the SIVEP-Gripe system: women who have recently given birth, heart disease, chronic hematological disease, Down syndrome, chronic liver disease, asthma, diabetes, chronic neurological disease, chronic lung disease, immunosuppression, chronic kidney disease, and obesity. Ventilatory support was classified as “Yes” (either invasive or non-invasive) or “No”.

### 2.3. Variables and Statistical Design

SARI cases from the SIVEP-Gripe database were categorized into four groups based on viral detection: (1) influenza—samples that tested positive for FLUAV and FLUBV only; (2) RSV—samples that tested positive for respiratory syncytial virus only; (3) COVID-19—samples that tested positive for SARS-CoV-2; and (4) Other respiratory viruses (OV)—samples that tested positive for all other respiratory viruses, including RV, HMPV, AdV, HBoV, and PIV. Statistical analysis was performed across three-time intervals: before the COVID-19 pandemic (2019), during the first year of the COVID-19 pandemic (2020), and for the entire study period (2019–2020). Categorical variables such as gender, city, self-reported ethnicity, clinical symptoms, clinical evolution, comorbidities, outcomes, and mechanical ventilation were presented as absolute frequencies (n), percentages (%), 95% confidence intervals (CIs), and odds ratios (OR) with respective *p*-values. Continuous variables (age and number of cases) were presented as individual values, mean ± standard deviation, interquartile range (IQR), median, and respective *p*-values. Age was normalized to years, and participants under 1 year old were grouped as zero (0). Comorbidities were recorded in the SIVEP-Gripe database as “Yes”, “Chronic”, and “No”, with individuals classified as “Chronic” reclassified as “Yes”. To minimize bias in symptom frequency assessment, patients with co-infections involving FLUV, RSV, or OV with SARS-CoV-2 were excluded from the analysis.

A total of 45,203 cases of SARI were notified by SIVEP-Gripe in Bahia State during 2019 and 2020, in which 34,425 (76.16%) underwent laboratory test and were therefore initially included in this study. Among the 34,425 submitted to laboratory testing, a total of 18,447 (53.58%) had a positive result by PCR for at least one of the eight respiratory viral agents analyzed (FLUV, RSV, SARS-CoV-2, RV, HMPV, AdV, HBoV, and PIV) and were therefore included in our study.

### 2.4. Population Age Pyramids and Overlapping Curves

Participants’ ages were stratified into the following groups: <10 years, 10–19 years, 20–29 years, 30–39 years, 40–49 years, 50–59 years, 60–69 years, 70–79 years, 80–89 years, and ≥90 years. Overlapping curves were generated to analyze age pyramid variations across different viruses and time periods using GraphPad Prism software (version 10.3.1, San Diego, CA, USA).

### 2.5. Statistical Analysis

Continuous variables were tested for normality using the Kolmogorov–Smirnov test with Lilliefors correction. Variables that did not meet normality assumptions were analyzed using the two-tailed Mann–Whitney U test and the Kruskal–Wallis test, followed by Dunn’s multiple comparison test. The association between categorical variables was assessed using the Chi-squared test and Fisher’s exact test. All statistical analyses were performed using GraphPad Prism software (version 10.3.1, San Diego, CA, USA). A significance level of 5% was used to determine statistical significance, with *p* < 0.05 considered significant.

## 3. Results

### 3.1. Characteristics of Study Population During 2019–2020

**Figure 1:**
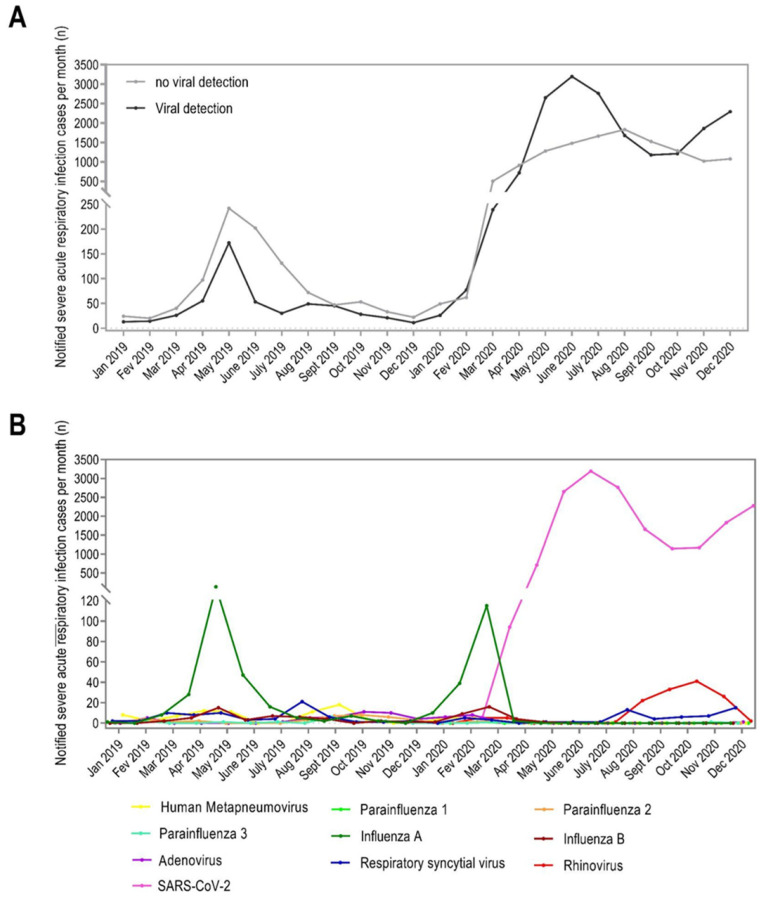
SARI notifications in Bahia State. Monthly notifications of SARI cases in Bahia State, Brazil, from January 2019 to December 2020, with confirmation by PCR. (**A**) All notification for SARI in Bahia state during January 2019 and December 2020, including those positive (viral detection, black line) and negative (no viral detection, gray line). (**B**) SARI notification by viruses during January 2019 and December 2020: human metapneumovirus (yellow), parainfluenza 1 (light green), parainfluenza 2 (orange), parainfluenza 3 (green), influenza A (green forest), influeza B (dark red), respiratory adenovirus (violet), respiratory syncytial virus (blue), rhinovirus (red), SARS-CoV-2 (pink).

During 2019 and 2020, the circulation of seven respiratory viruses associated with SARI was recorded as follows: SARS-CoV-2 (94.83%), FLUV (2.67%), RV (0.73%), RSV (0.68%), HMPV (0.47%), AdV (0.32%), and PIV (0.29%). Figure 1A represents the discrepancy between notification with viral and no viral detection. After screening our dataset, a total of 18,447 notifications met the inclusion and exclusion criteria for statistical analysis. General characteristics of the study population are presented in Table 1.

### 3.2. Shifts in Age Distribution of SARI Cases Are Associated with the Introduction and Circulation of SARS-CoV-2 in Bahia State

First, we analyzed the distribution of SARI cases during two periods: 2019 and 2020, pre- and post-SARS-CoV-2 introduction and dissemination. A closer inspection of these data reveals that while FLUV notifications decreased in March 2020, a peak in RV cases occurred after SARS-CoV-2 introduction in October 2020 (Figure 1B).

**Figure 2:**
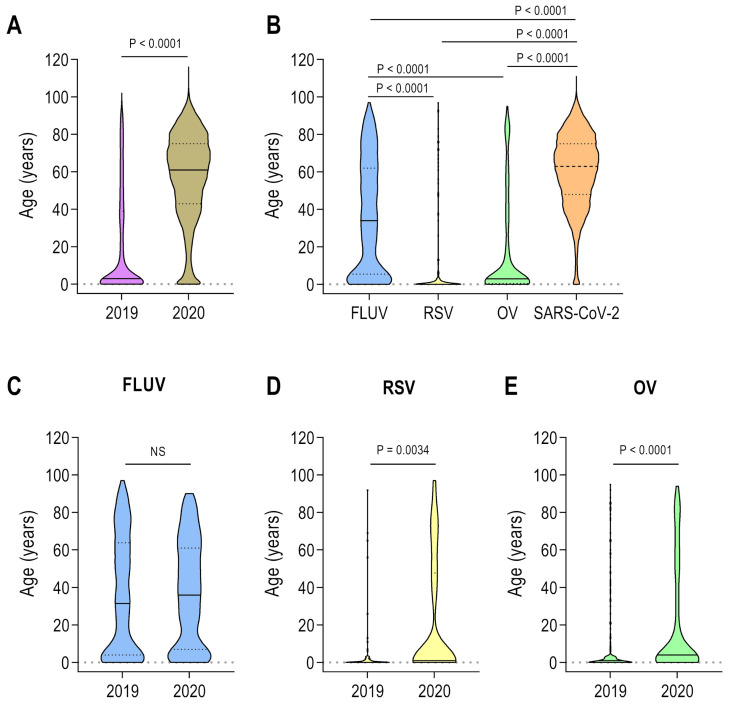
Age distribution of individuals with SARI caused by different respiratory viruses. (**A**) Age distribution of total SARI cases caused by respiratory viruses in 2019 and 2020. (**B**) Age distribution of total SARI cases stratified by specific viruses. (**C**) Age distribution of total SARI cases caused by influenza virus (FLUV). (**D**) Age distribution of total SARI cases caused by respiratory syncytial virus (RSV). (**E**) Age distribution of total SARI cases caused by other respiratory viruses (OV). The following statistical analyses were performed: Two-tailed Mann–Whitney U test (**A**,**C**–**E**) and Kruskal–Wallis test followed by Dunn’s multiple comparison test (**B**).

We also quantified the shift in age distribution among different viruses (Figure 2). SARI cases reported in 2020 were generally older than those reported in 2019 (mean age 57 vs. 20.6 years, respectively; *p* < 0.0001; Figure 2A). SARS-CoV-2 was more frequently reported in older individuals (mean age 60.6 years), FLUV in adults (mean age 36.1 years), other viruses (OV) in younger individuals (mean age 18.8 years), and RSV in children (mean age 12.4 years; Figure 2B). Notably, we observed a significant shift in the average age before and during the COVID-19 pandemic for RSV notifications (mean age 5.3 vs. 21.2 years, respectively; *p* = 0.0034; Figure 2D) and OV (mean age 11.5 vs. 21.2 years, respectively; *p* < 0.0001; Figure 2E) but no significant change for FLUV (mean age 35.4 vs. 37.1 years, respectively; *p* > 0.05; Figure 2C).

**Figure 3:**
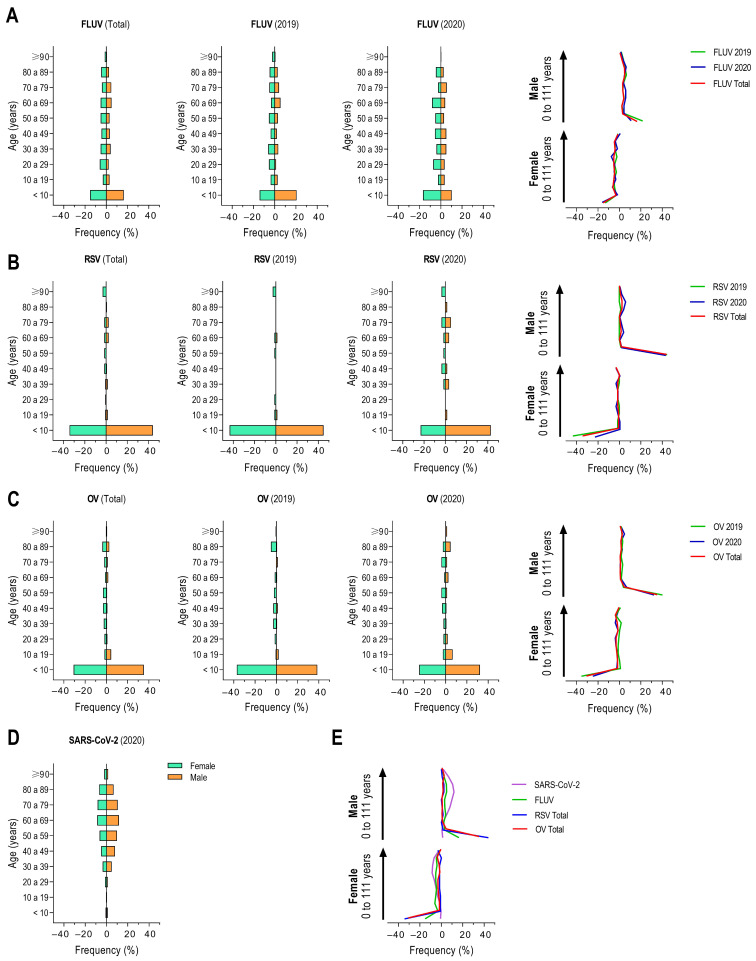
Population pyramid frequency of individuals with SARI caused by different respiratory viruses. Population pyramid frequency for influenza virus (FLUV) (**A**), respiratory syncytial virus (RSV) (**B**), other respiratory viruses (OVs) (**C**), and SARS-CoV-2 (**D**), with respective overlapping curves (on the right). (**E**) Overlapping curves for total SARI cases caused by respiratory viruses.

Pyramid frequency analysis confirmed these findings and suggested a shift in the gender distribution of notifications during the COVID-19 pandemic for FLUV, RSV, and OV (Figure 3). A modest change in the frequency of notifications from male to female was observed for FLUV (Figure 3A). RSV notifications were less frequent in females (Figure 3B), and OV notifications were less frequent in females during the COVID-19 pandemic compared to the pre-COVID-19 period (Figure 3C). These data support our hypothesis that the presence of COVID-19 marked a turning point in the epidemiology of respiratory viruses in Bahia, Brazil.

### 3.3. Circulation of SARS-CoV-2 Alters the Clinical-Epidemiological Characteristics of SARI Caused by FLUV, RSV, and OV

**Figure 4:**
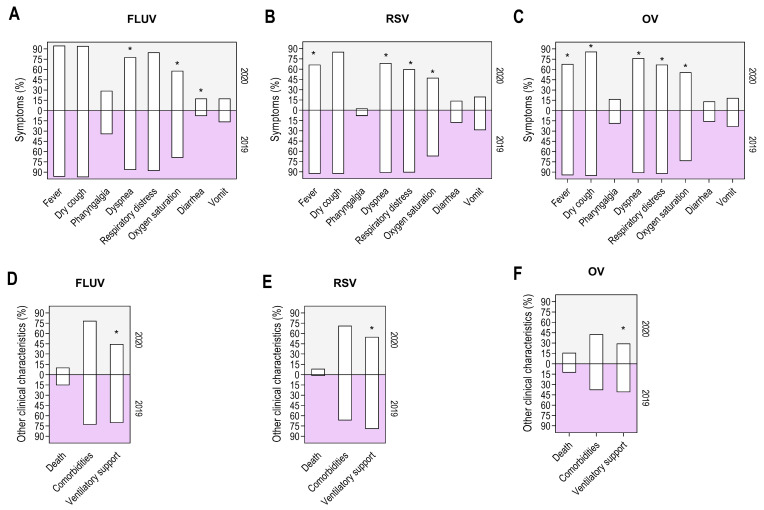
Differences in symptoms and other clinical characteristics of SARI by year reported in Bahia State, Brazil, during 2019–2020. Frequency of symptoms in individuals diagnosed with SARI according to infection caused by FLUV (**A**), RSV (**B**), and OVs (**C**). Frequency of other clinical characteristics (outcomes, comorbidities, and use of ventilatory support) in individuals diagnosed with SARI according to infection caused by FLUVs (**D**), RSV (**E**), and OVs (**F**). Asterisk (*) indicates statistical significance with *p* < 0.05 between 2019 x 2020.

To investigate the potential impact of SARS-CoV-2 introduction and dissemination on the clinical characteristics of other respiratory viruses, we analyzed the association between symptom frequency, interventions, and outcomes during two periods: pre- and post-COVID-19 pandemic. For FLUV and RSV, we observed a significant difference in symptom frequency, which was higher before the COVID-19 pandemic compared to during the pandemic (Figure 4A,B). However, this was not observed for OVs (Figure 4C). Additionally, the frequency of comorbidities and the need for ventilatory support decreased for FLUV and RSV during the COVID-19 pandemic (Figure 4D,E), while a slight variation was observed for OV (Figure 4F).

Although changes in average age and gender distribution may help explain the epidemiological shifts in these viruses, alterations in symptoms and clinical characteristics cannot be attributed solely to these factors or to the natural history of the viruses. To further investigate, we conducted univariate analyses to identify significant changes in symptoms before and during the COVID-19 pandemic and to evaluate whether the presence of SARS-CoV-2 increased the odds of specific symptoms (Table 2).

A closer examination of the data revealed that SARS-CoV-2 circulation had a significant impact on the symptom profile of OV, with 62% of evaluated symptoms showing modification from the pre-pandemic to the pandemic period. This was followed by RSV (50%) and FLUV (37%). Except for diarrhea, we observed an elevated odds ratio (OR) for symptoms reported in 2019 (pre-pandemic) compared to 2020 (during the pandemic; Table 2). In 2019, reported cases showed high ORs for dyspnea in FLUV (OR 1.8, *p* = 0.0138), RSV (OR 4.7, *p* = 0.0022), and OV (OR 3.1, *p* = 0.0006; Table 2), compared to 2020. Similarly, high ORs were observed for fever and respiratory distress in RSV (OR 6.5, *p* = 0.0004; OR 6.7, *p* = 0.0001, respectively) and OV (OR 8.2, *p* < 0.0001; OR 5.3, *p* < 0.0001, respectively). For OV, we also found high odds for dry cough (OR 2.6, *p* = 0.0290) and low oxygen saturation (OR 2.2, *p* = 0.0019) in 2019 cases compared to those during the pandemic. Interestingly, individuals before the pandemic were more likely to present with these symptoms, which was unexpected.

We analyzed clinical outcomes and the requirement for ventilatory support. SARS-CoV-2 circulation did not affect the mortality rates for FLUV, RSV, or OV. Furthermore, cases reported before the COVID-19 pandemic were more likely to require ventilatory support than those reported during the pandemic, with ORs for FLUV (OR 2.94, *p* < 0.0001), RSV (OR 3.15, *p* = 0.0086), and OV (OR 2.94, *p* = 0.0003; Table 3).

### 3.4. SARI Caused by SARS-CoV-2 Exhibits Distinct Clinical Characteristics and Outcomes Compared to Other Groups

**Figure 5:**
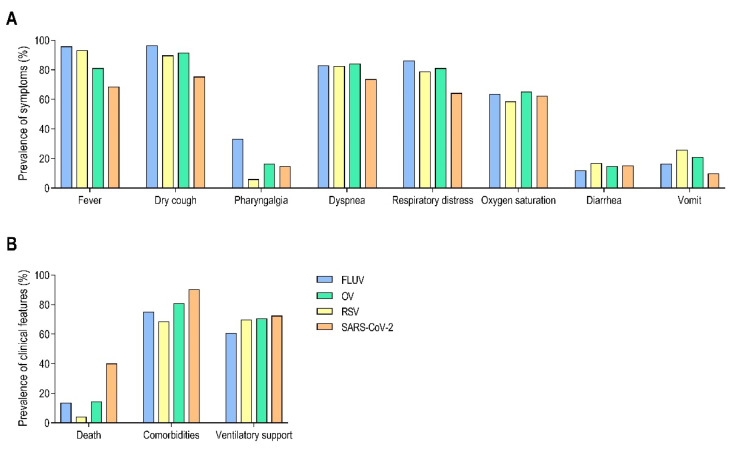
Prevalence of symptoms and other clinical characteristics of SARI by year reported in Bahia State, Brazil, during 2019–2020. Prevalence of symptoms (**A**) and other clinical characteristics (outcomes, comorbidities, and use of ventilatory support) (**B**) in cases with confirmed laboratory results for FLUV (blue), OV (green), RSV (yellow), and SARS-CoV-2 (orange).

A descriptive analysis of all groups (FLUV, RSV, SARS-CoV-2, and OV) revealed that differences in symptom profiles were present during the study period, not only between pre- and during-COVID-19 but also among the viruses themselves (Figure 5). SARS-CoV-2 exhibited a significantly lower frequency of fever, dry cough, dyspnea, respiratory distress, and vomiting compared to the other viruses (Figure 5A). However, high mortality and a greater prevalence of comorbidities were more common in SARS-CoV-2 cases than in the other groups (Figure 5B). Interestingly, the need for ventilatory support was similar across all viruses.

Using univariate analysis, we compared the symptom profiles among the respiratory viruses in our study (Table 4). RSV and OV showed similar symptom profiles despite differences in prevalence (*p* > 0.05). FLUV and SARS-CoV-2 differed in all symptoms except for oxygen saturation, which had a similar profile between the two viruses (*p* > 0.05). Overall, SARS-CoV-2 symptoms differed the most from FLUV (87.5%), RSV (87.5%), and OV (62.5%). FLUV, RSV, and OV had more comparable symptom profiles, with differences in only 50% to 0% of symptoms (Table 4). Notably, all viruses exhibited similar frequencies of altered oxygen saturation.

In line with these findings, we explored the impact of these symptom profiles on clinical outcomes. Individuals diagnosed with SARS-CoV-2 were more likely to die than those diagnosed with FLUV (*p* < 0.0001), RSV (*p* < 0.0001), and OV (*p* < 0.0001; Table 5). However, no significant differences were observed in the need for ventilatory support between SARS-CoV-2 and RSV (*p* = 0.5613) or OVs (*p* = 0.4793); a difference was only noted for FLUV (*p* < 0.0001). As expected, individuals with SARS-CoV-2 were more likely to have comorbidities compared to those with FLUVs (*p* < 0.0001), RSV (*p* < 0.0001), and OVs (*p* = 0.0003).

## 4. Discussion

Our study aimed to evaluate the impact of the introduction of SARS-CoV-2 on the epidemiology of other respiratory viruses in the state of Bahia, Brazil. We demonstrated that the introduction of SARS-CoV-2 during the COVID-19 pandemic altered the number of cases of influenza, respiratory syncytial virus, and other viral infections. Furthermore, individuals who tested positive for these viruses showed significant changes in the frequency of symptoms such as fever, oxygen saturation, and dry cough, as well as changes in the need for ventilatory support. The frequency of these symptoms was lower after the introduction of SARS-CoV-2 in 2020 compared to the previous period in 2019. Additionally, we observed a shift in the age group of individuals affected by respiratory viruses when comparing the periods before and after the circulation of SARS-CoV-2.

The impacts of the COVID-19 pandemic extend beyond the mortality rates of infected individuals and the economic burden associated with prevention, restrictions, and treatment measures [26,27]. Growing evidence suggests that long-term effects and syndromes are linked to post-COVID-19 conditions in individuals who have recovered from the infection [28,29]. Recent analyses also indicate that the introduction and spread of SARS-CoV-2 have had an epidemiological impact on infections caused by other respiratory viruses [17,21]. It has been suggested that the seasonality of respiratory viral infections has been directly altered, raising new questions regarding the dynamics and incidence of post-COVID-19 respiratory infections, the role of children and adults in transmitting these viruses, and potential changes in symptom profiles and clinical conditions [30,31].

Our data show that after the emergence of SARS-CoV-2 in early 2020, cases of SARI caused by FLUV and RSV decreased, which was expected given the emergency strategies implemented to contain the spread of COVID-19. Following the emergence of SARS-CoV-2, there was a resurgence of RSV and RV cases, whereas FLUV cases did not increase during the studied period. Notably, an increase in RSV and RV positivity was observed in São Paulo, Brazil, in 2021 and 2022, while FLUV cases rose more gradually [32].

Students represent a significant group affected by respiratory diseases. Research by Billard and colleagues highlighted international shifts in RSV epidemiology due to school closures and stay-at-home policies. Specifically, a 39-week delay in the RSV season was observed in 11 countries during COVID-19, followed by a resurgence as schools reopened and stay-at-home mandates were lifted [33]. Similarly, we observed a resurgence of RSV and RV in Bahia State, as well as similar trends in southern Brazil following the introduction of COVID-19 [34]. While a decline in FLUV cases during COVID-19 has been well-documented [35,36,37], some regions worldwide have experienced a resurgence of FLUV activity during and after the pandemic, covering both the 2021–2022 and the 2022–2023 seasons [38,39,40,41,42,43,44,45,46,47]. A substantial decrease in the incidence and prevalence of different infectious diseases (other than those caused by respiratory viruses) was observed in 2020 [48]. However, an increased incidence in many other infectious diseases such as syphilis and dengue has been observed after a relaxation of public health measures for containing COVID-19 during the pandemic period [49,50].

Before the availability of vaccines, the elderly population exhibited high SARS-CoV-2 positivity and mortality rates, whereas SARI caused by other respiratory viruses primarily affected younger individuals. In our study, we found that SARI cases reported in 2020 involved older individuals compared to those reported in 2019. Interestingly, SARS-CoV-2 altered the age distribution of reported RSV and OV cases, but not FLUV. A systematic review indicated a 79.0% reduction in RSV-related hospitalizations in high-income countries and a 13.8% reduction in low-income countries among children aged 0 to 60 months during COVID-19, supporting our findings [51]. Both FLUV and RSV cases showed an increased average age in some regions during the pandemic [17]. Beyond age distribution, we unexpectedly found that individuals pre-pandemic were more likely to exhibit symptoms.

Recent studies suggest that SARS-CoV-2 has not only impacted respiratory diseases but also influenced the epidemiology of cardiovascular diseases and bacterial infections [52,53]. In our study, we observed changes in the symptom profiles of FLUV, RSV, and OV when comparing pre- and post-COVID-19 periods. FLUV patients were more likely to exhibit dyspnea and diarrhea before the pandemic. While COVID-19 reduced the incidence of SARI associated with these viruses, it was unexpected that these infections would present with fewer symptoms. Notably, FLUV patients showed variations in only two symptoms. Among common symptoms—fever, sore throat, rhinorrhea, headache, cough, and myalgia—FLUV cases exhibited a high frequency of symptoms, except for fever, which was more frequent in COVID-19 patients [54].

Before the pandemic, RSV patients exhibited higher odds for fever, dyspnea, and respiratory distress. A study by Bhardwaj and colleagues in India not only documented a resurgence of RSV but also reported shifts in symptoms such as fever, sore throat, breathlessness, and cough from the pre-pandemic period (2016 to January 2020) to the pandemic period (2020–2023) [55]. This suggests that the frequency of symptoms may vary by region. Additionally, one may wonder if changes in symptom frequency have influenced mortality profiles. In our study, we found that patients with FLUV, RSV, and OV infections reported prior to the COVID-19 pandemic required ventilatory support more frequently than those during the pandemic. However, the circulation of SARS-CoV-2 did not appear to affect the outcomes of FLUV, RSV, or OV infections.

As suggested by Pinotti and colleagues, the impact of new viral strains on the epidemiology of other viruses may emerge gradually, sometimes years after their initial introduction [56]. The emergence of SARS-CoV-2 seems to have had early effects on the epidemiology of respiratory viruses. Further analysis, incorporating updated data and mathematical models, could provide a clearer understanding of both the immediate and long-term impacts.

Our study presents some limitations. As it is an observational study using secondary data from government health databases, it is necessary to highlight the difficulties related to delays in notifications within the systems, the unavailability of records, and data transfers, as well as the presence of underreporting, which may lead to data incompleteness. This can limit the scope of the data obtained. However, it is important to emphasize the relevance of disease monitoring systems, such as SIVEP-Gripe and the Sistema de Informação de Agravos de Notificação (SINAN) in Brazil, as they are crucial for the development of public policies. The data obtained from observational studies and secondary databases serve as the foundation for conducting more complex epidemiological studies. Since SIVEP-Gripe is a nationwide database, the data used in this study provide a broad population-based perspective, enabling a more robust analysis of the reality and impact of respiratory diseases in the state of Bahia, which faces low Human Development Index (HDI) levels and economic, social, and environmental challenges.

## 5. Conclusions

In conclusion, our findings suggest that the emergence and spread of SARS-CoV-2 have significantly influenced respiratory viral epidemiology in Bahia State, Brazil, leading to changes in age distribution, symptom profiles, and viral positivity rates. Notably, SARS-CoV-2 has impacted the frequency of symptoms such as fever, dyspnea, dry cough, and respiratory distress among infections caused by influenza, respiratory syncytial virus, and other respiratory viruses. Additionally, shifts in the age profile of infected individuals highlight evolving patterns of vulnerability. These insights underscore the need for ongoing evaluations of health policies to address the changing landscape of respiratory viral infections.

## Figures and Tables

**Table 1:** Characteristics of the studied population from 2019 to 2020.

	**Median**	**Q1–Q3**
Age, years	62	46–75
	**n**	**%**
Gender		
Female	10,199	55.47
Male	8187	44.53
Self-declared ethnicity		
Yellow	142	0.77
White	1684	9.13
Indigenous	25	1.35
Brown	9531	51.67
Black	1331	7.10
Not informed	5575	30.22
SIVEP-Gripe Notification positive by PCR	18,447	100
Influenza virus (FLUV)	493	2.67
Parainfluenza (PIV)	54	0.29
Respiratory syncytial virus (RSV)	126	0.68
Adenovirus (AdV)	59	0.32
Human metapneumovirus (HMPV)	87	0.47
Bocavirus (HBoV)	0	0.00
Rhinovirus (RV)	135	0.73
Severe acute respiratory syndrome coronavirus 2 (SARS-CoV-2)	17,493	94.83

**Table 2:** Association between symptoms frequency and time periods of study (pre-COVID-19 pandemic and the first year of COVID-19 pandemic) for respiratory virus infection ^€^.

	Influenza Virus (FLUV)				Respiratory Syncytial Virus (RSV)				Other Respiratory Viruses (OV)			
Symptoms ^†^	2019 N = 296 (%)	2020 N = 190 (%)	OR (CI 95%)	*p* ^‡^	2019 N = 70 (%)	2020 N = 45 (%)	OR (CI 95%)	*p* ^‡^	2019 N = 150 (%)	2020 N = 159 (%)	OR (CI 95%)	*p* ^‡^
Fever												
Yes	282 (96.6)	175 (95.1)	1.450 (0.60–3.67)	0.4261	65 (92.8)	26 (66.7)	6.500 (2.02–17.51)	**0.0004**	142 (94.6)	95 (68.3)	8.221 (3.66–17.35)	**<0.0001**
No	10 (3.4)	9 (4.9)			5 (7.2)	13 (33.3)			8 (5.4)	44 (31.7)		
Not informed *	4	6			0	6			0	20		
Dry cough												
Yes	286 (97.3)	172 (94.5)	2.078 (0.79–5.23)	0.1232	64 (92.8)	35 (85.4)	2.194 (0.57–6.93)	2117	141 (95.3)	128 (86.2)	2.675 (1.12–7.00)	**0.0290**
No	8 (2.7)	10 (5.5)			5 (7.2)	6 (14.6)			7 (4.7)	17 (13.8)		
Not informed *	2	8			1	4			2	14		
Pharyngalgia												
Yes	80 (34.5)	46 (29.1)	1.233 (0.79–1.90)	0.3465	4 (8.2)	1 (2.8)	3.111 (0.47–39.02)	0.3902	21 (19.3)	19 (17.1)	1.156 (0.58–2.32)	0.6795
No	158 (65.5)	112 (70.9)			45 (91.8)	35 (97.2)			88 (80.7)	92 (82.9)		
Not informed *	58	32			21	9			41	48		
Dyspnea												
Yes	250 (86.8)	139 (78.1)	1.846 (1.14–2.98)	**0.0138**	64 (91.4)	29 (69.0)	4.782 (1.63–13.99)	**0.0022**	135 (91.2)	111 (76.5)	3.181 (1.62–6.52)	**0.0006**
No	38 (13.2)	39 (21.9)			6 (8.6)	13 (31.0)			13 (8.8)	34 (24.5)		
Not informed *	8	12			0	3			2	14		
Respiratory distress												
Yes	258 (88.3)	153 (85.0)	1.339 (0.76–2.28)	0.2911	61 (91.0)	24 (60.0)	6.778 (2.43–19.52)	**0.0001**	134 (92.4)	95 (67.2)	5.386 (2.68–11.08)	**<0.0001**
No	34 (11.7)	27 (15.0)			6 (9.0)	16 (40.0)			11 (7.6)	42 (32.8)		
Not informed *	4	10			3	5			5	22		
Oxygen saturation												
Yes	195 (69.1)	98 (58.3)	1.601 (1.08–2.37)	**0.0199**	43 (67.2)	20 (47.6)	2.007 (0.99–4.86)	**0.0448**	101 (73.7)	77 (56.0)	2.223 (1.32–3.74)	**0.0019**
No	87 (30.9)	70 (41.7)			21 (32.8)	22 (52.4)			36 (26.3)	61 (44.0)		
Not informed *	14	22			6	3			13	21		
Diarrhea												
Yes	21 (8.0)	29 (17.7)	0.405 (0.22–0.74)	**0.0026**	10 (18.2)	5 (13.9)	1.378 (0.46–3.92)	0.5397	21 (16.5)	16 (13.4)	1.275 (0.63–2.53)	0.4981
No	241 (92.0)	135 (82.3)			45 (81.8)	31 (86.1)			106 (84.5)	103 (86.6)		
Not informed *	34	26			15	9			23	40		
Vomit												
Yes	45 (17.0)	28 (17.7)	0.950 (0.57–1.60)	0.8454	16 (29.1)	8 (20.0)	1.641 (0.65–4.37)	0.3140	31 (23.8)	22 (18.5)	1.381 (0.74–2.56)	0.3021
No	220 (83.0)	130 (82.3)			39 (70.9)	32 (80.0)			99 (76.2)	97 (81.5)		
Not informed *	31	32			15	5			20	40		

^€^ To avoid bias in the analysis of symptoms in viral SARS cases, we excluded patients with SARS-CoV-2 co-infection during the association analysis. ^†^ Patients whose respective symptoms were not reported were not considered for statistical analysis. ^‡^ Chi-square test and Fisher’s Exact test. * Not included in statistical analysis. Abbreviations: OR, Odds ratio; CI 95%, Confidence interval 95%.

**Table 3:** Clinical characteristics and outcomes of individuals diagnosed with SARI in Bahia State, Brazil, during 2019–2020.

	Outcome	2019—n (%)	2020—n (%)	OR	CI 95%	*p* Value ^‡^
Influenza virus	Death	38 (15.5)	18 (10.6)	1.540	0.84–2.78	0.1553
	Cure	207 (84.5)	151 (89.4)			
	Not informed *	51	21			
	**Comorbidities**					
	Yes	114 (73.1)	70 (78.6)	0.737	0.41–1.36	0.3318
	No	42 (26.9)	19 (21.4)			
	Not informed *	140	101			
	**Ventilatory support**					
	Yes	188 (70.4)	72(44.7)	2.942	1.96–4.36	**<0.0001**
	No	79 (29.6)	89 (55.3)			
	Not informed *	29	29			
Respiratory syncytial virus	**Outcome**					
	Death	1 (1.7)	3 (8.8)	0.181	0.01–1.28	0.2792
	Cure	57 (98.3)	31 (91.2)			
	Not informed *	12	11			
	**Comorbidities**					
	Yes	12 (66.7)	10 (71.4)	0.800	0.21–3.48	>0.9999
	No	6 (33.3)	4 (28.6)			
	Not informed *	52	31			
	**Ventilatory support**					
	Yes	50 (79.4)	22 (55.0)	3.147	1.32–7.21	**0.0086**
	No	13 (20.6)	18 (45.0)			
	Not informed *	7	5			
Other viruses	**Outcome**					
	Death	15 (12.8)	21 (15.9)	0.777	0.38–1.60	0.4891
	Cure	102 (87.2)	111 (84.1)			
	Not informed *	33	26			
	**Comorbidities**					
	Yes	50 (38.2)	57 (42.7)	0.689	0.29–1.70	0.4037
	No	14 (10.7)	11 (8.4)			
	Not informed *	86	91			
	**Ventilatory support**					
	Yes	113 (41.1)	81 (29.4)	2.940	1.73–5.09	**<0.0001**
	No	28 (10.2)	59 (19.3)			
	Not informed *	9	19			

Patients whose respective outcome, comorbidity and ventilatory support requirement were not reported were not considered for statistical analysis. ^‡^ Chi-square test and Fisher’s Exact test. Abbreviations: OR, Odds ratio; CI 95%, Confidence interval 95%. * Not included in statistical analysis.

**Table 4:** Clinical difference in symptoms between viruses causing SARI, reported in the State of Bahia, Brazil.

Symptoms ^†^	Group	Prevalence (n)	FLUV × RSV	FLUV × OV	FLUV × SARS-CoV-2	RSV × OV	RSV × SARS-CoV-2	OV × SARS-CoV-2
**Fever**	FLUV	96.0 (457/476)	***p* < 0.0001**	***p* < 0.0001**	***p* < 0.0001**	*p* = 0.7296	***p* = 0.0009**	***p* < 0.0001**
	RSV	83.5 (91/109)						
	OV	82.0 (237/289)						
	SARS-CoV-2	68.7 (10,571/15,388)						
**Dry cough**	FLUV	96.2 (458/476)	***p* = 0.0067**	***p* = 0.0090**	***p* < 0.0001**	*p* = 0.5657	***p* = 0.0004**	***p* < 0.0001**
	RSV	90.0 (99/110)						
	OV	91.8 (269/293)						
	SARS-CoV-2	75.3 (11,866/15,747)						
**Pharyngalgia**	FLUV	31.8 (126/396)	***p* < 0.0001**	***p* = 0.0003**	***p* < 0.0001**	***p* = 0.0066**	***p* = 0.0217**	*p* = 0.1516
	RSV	5.9 (5/85)						
	OV	18.2 (40/220)						
	SARS-CoV-2	14.7 (1913/12,994)						
**Dyspnea**	FLUV	83.5 (389/466)	*p* = 0.9104	*p* = 0.8610	***p* < 0.0001**	*p* = 0.8219	***p* = 0.0247**	***p* < 0.0001**
	RSV	83.0 (93/112)						
	OV	84.0 (246/293)						
	SARS-CoV-2	73.7 (11,715/15,904)						
**Respiratory** **distress**	FLUV	87.1 (411/472)	***p* = 0.0418**	***p* = 0.0295**	***p* < 0.0001**	*p* = 0.6933	***p* = 0.0011**	***p* < 0.0001**
	RSV	79.4 (85/107)						
	OV	81.2 (229/282)						
	SARS-CoV-2	64.3 (9814/15,272)						
**Oxygen** **saturation**	FLUV	65.1 (293/450)	*p* = 0.2732	*p* = 0.9163	*p* = 0.2654	*p* = 0.3369	*p* = 0.5113	*p* = 0.4563
	RSV	59.4 (63/106)						
	OV	64.7 (178/275)						
	SARS-CoV-2	62.5 (9390/15,016)						
**Diarrhea**	FLUV	9.4 (50/426)	*p* = 0.2151	*p* = 0.2191	***p* = 0.0386**	*p* = 0.7448	*p* = 0.7756	*p* = 0.8767
	RSV	16.5 (15/91)						
	OV	15.0 (37/246)						
	SARS-CoV-2	15.4 (2046/13,285)						
**Vomit**	FLUV	17.2 (73/423)	*p* = 0.0707	*p* = 0.1964	***p* < 0.0001**	*p* = 0.4287	***p* < 0.0001**	***p* < 0.0001**
	RSV	25.3 (24/95)						
	OV	21.3 (53/249)						
	SARS-CoV-2	9.7 (1283/13,186)						

^†^ Patients whose respective symptoms were not reported were not considered for statistical analysis. Analyzed by Chi-square test.

**Table 5:** Difference in clinical characteristics and outcomes of individuals between viruses causing SARI, reported in the State of Bahia, Brazil.

Clinical Features ^†^	Group	Variable		FLUV × RSV	FLUV × OV	FLUV × SARS-CoV-2	RSV × OV	RSV × SARS-CoV-2	OV × SARS-CoV-2
		Death n (%)	Cure n (%)	*p* Value ^‡^					
**Outcome**	FLUV	56 (13.5)	358 (86.5)	***p* = 0.0119**	*p* = 0.7369	***p* < 0.0001**	***p* = 0.0080**	***p* < 0.0001**	***p* < 0.0001**
	RSV	4 (4.3)	88 (95.7)						
	OV	36 (14.4)	213 (85.6)						
	SARS-CoV-2	6279 (40.1)	9367 (59.9)						
		**Yes** **n (%)**	**No** **n (%)**	***p* value ^‡^**					
**Comorbidities**	FLUV	184 (75.1)	61 (24.9)	*p* = 0.4389	*p* = 0.1884	***p* < 0.0001**	*p* = 0.1273	***p* < 0.0001**	***p* = 0.0003**
	RSV	22 (68.7)	10 (31.3)						
	OV	107 (81.1)	25 (18.9)						
	SARS-CoV-2	11,239 (90.3)	1211 (9.7)						
		**Yes** **n (%)**	**No** **n (%)**	***p* value ^‡^**					
**Ventilatory** **support**	FLUV	260 (60.7)	168 (39.3)	*p* = 0.0848	***p* = 0.0244**	***p* < 0.0001**	*p* = 0.8709	*p* = 0.5580	*p* = 0.4743
	RSV	72 (69.9)	31 (31.1)						
	OV	194 (69.0)	87 (31.0)						
	SARS-CoV-2	11,549 (72.5)	4383 (27.5)						

^†^ Patients whose respective clinical features were not reported were not considered for statistical analysis. ^‡^ Chi-square test and Fisher’s Exact test.

## Data Availability

The data presented in this study are available on request from the corresponding authors due to ethical reasons including data from humans subjects.
